# Rehabilitation of a One-day-Old Neonate with Cleft Lip and Palate using Palatal Obturator: A Case Report

**DOI:** 10.5005/jp-journals-10005-1154

**Published:** 2012-08-08

**Authors:** Rajesh Bansal, Ashish Kumar Pathak, Baldev Bhatia, Sailesh Gupta, Keshav Kumar Gautam

**Affiliations:** Assistant Professor, Faculty of Dental Sciences, Institute of Medical Sciences, Banaras Hindu University, Varanasi-221005, Uttar Pradesh India, e-mail: rajeshbansal97@rediffmil.com; Student, Dental Mechanics, Faculty of Dental Sciences, Institute of Medical Sciences, Banaras Hindu University, Varanasi, Uttar Pradesh India; Professor and Dean, Department of Pediatric Medicine, Faculty of Medicine, Institute of Medical Sciences, Banaras Hindu University Varanasi, Uttar Pradesh, India; Assistant Professor, Department of Human Physiology, Faculty of Medicine, Institute of Medical Sciences, Banaras Hindu University Varanasi, Uttar Pradesh, India; Senior Resident, Faculty of Dental Sciences, Institute of Medical Sciences, Banaras Hindu University, Varanasi, Uttar Pradesh, India

**Keywords:** Cleft lip palate, Infant, Impression, Obturator, Presurgical orthopedics

## Abstract

Feeding a neonate with a complete cleft lip and palate is difficult pursuit due to communication between oral cavity and nasal cavity. A multidisciplinary approach is required to manage the complex problems involved in case of such neonates and their families. Present case is of a 1-day-old neonate having complete bilateral cleft lip and palate for which palatal obturator was constructed. A stepwise simple, easy and uncomplicated procedure for making accurate impressions, maxillary cast and fabrication of palatal obturator in infants with cleft lip and palate has been presented. The objective to present this case report is to emphasize the fact that how these palatal obturators /plates help in feeding, speech/language development, presurgical orthopedics and prevent other associated otorhinolaryngeal problems.

**How to cite this article:** Bansal R, Pathak AK, Bhatia B, Gupta S, Gautam KK. Rehabilitation of a One-day Old Neonate with Cleft Lip and Palate using Palatal Obturator: A Case Report. Int J Clin Pediatr Dent 2012;5(2):145-147.

## INTRODUCTION

Cleft lip and palate (CLP) is the most common congenital defect involving the orofacial region.^1^ Its presence may be solitary or it may be associated with congenital heart defects, skeletal anomalies, ocular lesions and mental retardation. The oronasal communication leads to nasal regurgitation and difficulty in suckling that complicates the feeding process. The aim of treatment in CLP patients is to restore normal anatomy and function. The crucial initial step in fabrication of obturator is the impression procedure.^2,3^ Thus, in this case report, stepwise procedure for fabrication of palatal obturator to rehabilitate the CLP patient has been presented to emphasize the fact that how these palatal obturators/plates help in feeding, speech/language development, presurgical orthopedics and prevent other associated otorhinolaryngeal problems.

## CASE REPORT

**Fig. 1:**
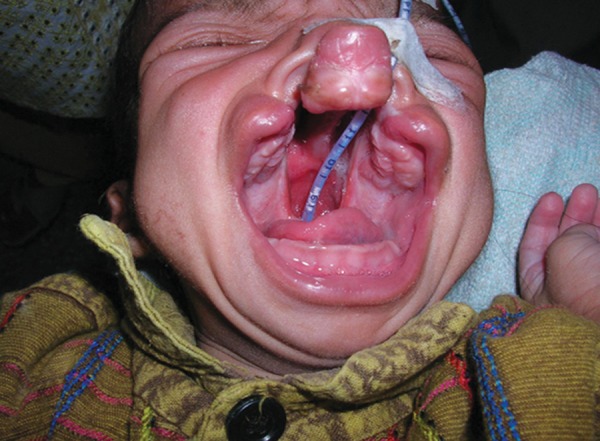
Intraoral view of a neonate with complete bilateral CLP

A 1-day-old male neonate with complete bilateral CLP (Fig. 1) was referred to the OPD of Unit of Prosthodontics, Faculty of Dental Sciences, Institute of Medical Sciences, Banaras Hindu University, Varanasi, India. First of all, counseling of the parents was done to reduce the emotions and anxiety. There was no family history suggestive of cleft lip and palate or teratogenic drug intake. Examination revealed that the patient had no other associated anomaly. The patient was being fed with the nasogastric intubation. The patient was fed only 1 hour earlier so he was posted for impressions next day. Parents were instructed not to feed the neonate for at least 2 hours prior to the procedure to avoid the regurgitation and aspiration. Stepwise procedure followed for fabrication of palatal obturator is described under.

**Fig. 2:**
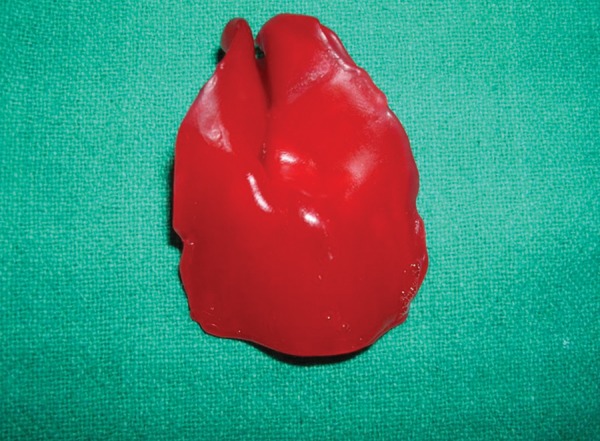
Wax sheet after initial intraoral adaptation

**Fig. 3:**
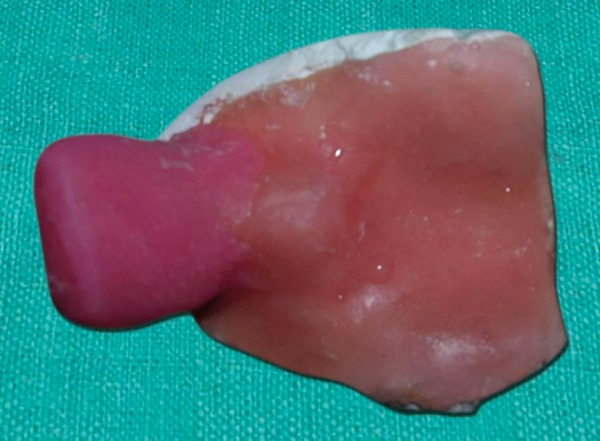
Custom acrylic tray with a handle is prepared on primary cast

A wax sheet of approximate size and shape was tailored and warmed before adapting intraorally. As soon as the wax was placed in the oral cavity with index and middle finger the baby started suckling during this period the wax was molded to the palate, taking care that the wax reaches to the mucobuccal fold areas. The wax mould was removed from mouth with minimum of distortion (Fig. 2). The plaster of paris cast was prepared to fabricate a custom impression trey (Fig. 3).

**Fig. 4:**
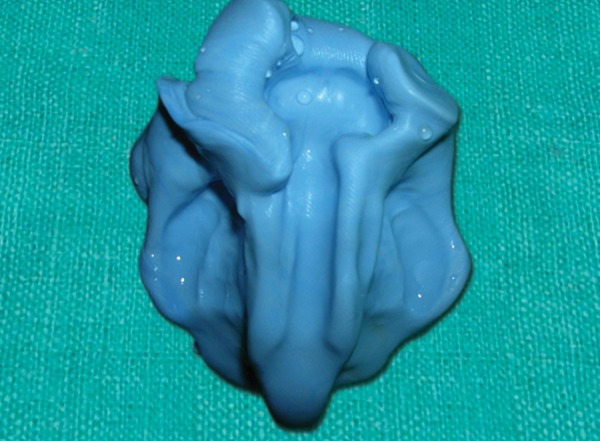
Final impression

The custom-made impression trey was evaluated intraorally to determine the easiest path of insertion and withdrawal. For making the impression the patient was taken to the neonatal intensive care unit with a pediatrician present at all the times to avoid complications and to handle airway emergencies. High volume suction was also kept ready, all the times, in case regurgitation of the stomach contents happens or impression material falls back into the airway during the procedure. Vinyl polysiloxane adhesive was applied over the intaglio surface and trey was loaded with vinyl polysiloxane impression material. The trey was carried to the mouth, placed in position and molding of the borders was done. The impression was made with the infant in fully awake condition. The infant was made to lie in a supine position on the lap of the parent with the head on the knee at a lower level. The clinician positioned himself in a comfortable 10 o'clock position to the infant's head. Monitoring of the baby's oxygen level throughout the impression making process was done to prevent accidental hypoxia. The baby was making the suckling movement and nasal breathing throughout the procedure. The impression was withdrawn with a snap movement after the setting of the impression material (Fig. 4).

**Fig. 5:**
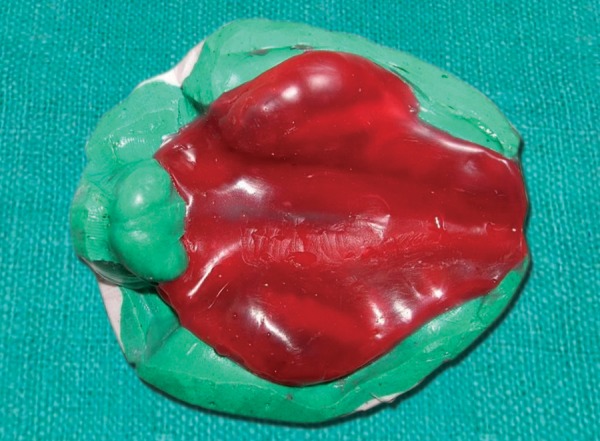
Wax pattern prepared on master cast

The impression was poured with the type V dental stone. The cast was inspected for the undercuts and blocked with clay. Then wax pattern was prepared with the help of pink color modeling wax (Fig. 5). Flasking, dewaxing, packing and curing were done in conventional manner.

**Fig. 6:**
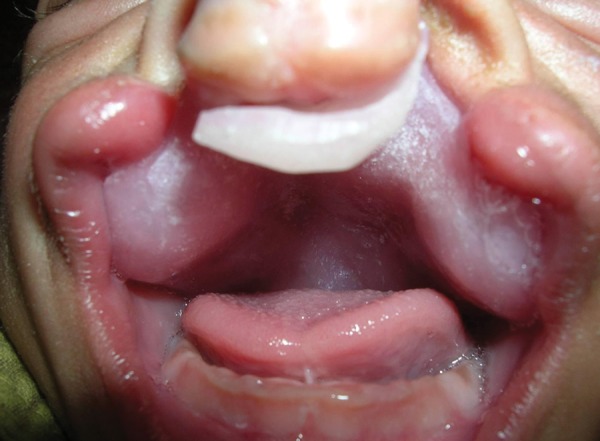
Intraoral view after insertion of prosthesis

After retrieval the prosthesis was inspected for uniform thickness, sharp edges, spicules and nodules; was modified as per need. After finishing and polishing the prosthesis was checked intraorally. Intaglio surface of the obturator was evaluated intraorally with the help of disclosing and delivered to the patient (Fig. 6). The parents were educated regarding the oral hygiene of prosthesis and the oral cavity.

## DISCUSSION

CLP runs in families and predilection for some races has also been documented. CLP is present approximately 1 in 750 live births, 0.133%. Male child has two times more predilection for CLP compared to female.^4,5^ Although there was no family history of any cleft in this case.

According to literature, there are various ways by which the problem of feeding may be tackled, i.e. (1) Specially designed nipples with wide openings that can increase the ejection of milk with reduced effort. (2) Orogastric or nasogastric tubes may be used but for a limited length of time. (3) presurgical infant maxillary orthopedics (PIMO) may prove to be beneficial to the surgeon, if a better alignment and closer approximation of the cleft segments is achieved before the actual surgical repair. (4) Surgery may close the communication but it may not prove to be beneficial in all cases, specially when the separation between the cleft segments is large (5) palatal obturator that is a definite help to the feeding of an infant.^6^ Obturator can also help in speech and language development but unfortunately little or no work has been done in this area.

Impression procedure is critical in the fabrication of obturator and should be carried out in the presence of pediatrician in the neonatal intensive care unit. Patient positioning, tray and impression material selection are the important factors to consider. A number of different positions of the infant have been adopted for CLP impression making in infants including face down, upright, horizontal raised to sitting as the impression sets and even inverted upside down.^7^ Various impression materials like alginate, low fusing impression compound and elastomeric (rubber base) impression materials have been routinely employed for making impression of neonate with CLP. The alginate material is not suitable for making these impressions as this has potential to tear due to low tear strength. Low fusing impression compound is a thermoplastic material, some times it can cause burn and volatile contents are released from this which have potential to be health hazard to the neonate.^8,9^ In our experience, elastomeric impression materials are better suited in making of cleft impression and they do not lead to any complications; due to its good elastic behavior, high tear strength, accurate reproduction of surface details and long-term dimensional stability which allows multiple pour. There are several biomaterials available for the maxillofacial prosthetics; like acrylic resins, visible light cured acrylic, acrylic polymer, silicones, etc. But, as yet, no material has emerged that possesses all the distinct and desirable properties.^10^ Acrylic resin was selected for making obturator as this is easily available and has good strength and can be fabricated with thin margin.

In summary, this article presents an easy to do and minimal risk procedure for the fabrication of palatal obturator for 1-day-old neonate. The team concept remains instrumental to the success in the care of these patients. Palatal obturator can prove to be helpful in minimizing the feeding problems, speech and language development in CLP patients.
